# Reconstruction plates versus single or dual miniplates for angle fractures: Hardware failure and non-union rates

**DOI:** 10.6026/973206300221250

**Published:** 2026-02-28

**Authors:** Mansi Dubey, Abhishek Balani, Vinay Kharsan, Abhishek Karan, Swati Sahu, Subham Mahapatra

**Affiliations:** 1 Department of Oral and Maxillofacial Surgery, New Horizon Dental College and Research Institute, Bilaspur, Chhattisgarh, India

**Keywords:** Mandibular angle fracture, reconstruction plates, miniplates, hardware failure, non-union, osteosynthesis

## Abstract

Mandibular angle fractures remain common in facial trauma, yet optimal fixation methods remain controversial due to variable hardware failure and non-union rates. Therefore, it is of interest to evaluate reconstruction plates (n=58), single miniplates (n=64) and dual miniplates (n=64) in 186 patients over 5 years with ≥12-month follow-up. Outcomes assessed included hardware failure, non-union, infection and malocclusion across all groups. Reconstruction plates demonstrated superior results with the lowest hardware failure (3.4%) and non-union (1.7%) rates versus single miniplates (12.5%, 6.3%) and dual miniplates (6.3%, 3.1%) (p<0.05). Reconstruction plates provide optimal biomechanical stability for complex mandibular angle fractures, particularly with unfavourable patterns, inadequate dentition, or poor patient compliance.

## Background:

Mandibular fractures form a significant percentage of the number of maxillofacial injuries that are experienced in clinical practice and the mandibular angle is one of the most common sites of anatomy that is vulnerable because of its structural susceptibility [1]. The root of the termination between the tooth-bearing mandibular body and the ascending ramus forms a biomechanically weak region that is prone to fracture propagation, especially in the presence of third molars [2]. According to epidemiological studies, it is always shown that angle fractures make up about 25-35% of all mandibular fractures and this is a big clinical dilemma for oral and maxillofacial surgeons [3]. The fracture management of the mandibular angle has continued to change significantly over the decades, from closed reduction and maxillomandibular fixation to the different open reduction and internal fixation methods [4]. When Champy *et al.* introduced miniplate osteosynthesis, the concept of the tension band fixation of the mandible became functional and thus the concept of the tension band became functional, which was termed the concept of functionally stable fixation [5]. It is a placement technique that promoted the introduction of a single miniplate on the external oblique ridge and where biomechanical principles are used to neutralise tensile forces during functional loading. The technical simplicity of this principle and the good results of monocortical fixation with one miniplate placed in the high mandibular border made the Champy principle widely accepted [6]. Nevertheless, the issue of biomechanical stability, especially in poor fracture types and those who do not respond to therapy, stimulated the discussion of other fixation options such as dual miniplate designs and load-bearing reconstruction plates [7]. Dual miniplate fixation, which involves the use of plates at the superior and inferior borders of the mandible, in theory, offers better three-dimensional stability since both tensile and compressive forces at the fracture site would be addressed [8]. Advocates believe that it eliminates micro-motion during functioning, hence enhancing the optimal healing of bones and also reducing the hardware- related complications. Nonetheless, the necessity of supplementary hardware, prolonged surgical dissection and possible neurovascular damage are the counterarguments to the regular implementation.

The reconstruction plates are the hardest fixation mode that can be offered to mandibular fractures to offer load-bearing but not load-sharing qualities [9]. These are bicortical screw-fixed and more robust plates of a greater thickness and are better mechanically stable, which entails transfacial or prolonged intraoral surgeries and results in risks of facial scarring and injury to the marginal mandibular nerve. Conventionally, reconstruction plates were only used with comminuted fractures, edentulous segments and pathologic needs, but recent findings indicate possible advantages in the implementation of angle fractures in routine [10]. Hardware failure, including plate fracture, loosening of screws and fixation loss, is a major complication after the fixation of a mandibular fracture [11]. The reported failure rates of the studies differ significantly with the fixation technique used the patient factors and the nature of the fracture reported, with a range of 2 to 15%. Hardware failure requires re-operation, further treatment and leads to the high cost of healthcare spending and putting patients at risk of further surgical risks The non-union, which refers to the inability to consolidate the bones in the anticipated healing periods, is seen in about 2-8 per cent of mandibular trauma and has a considerable effect on the quality of life of patients [12]. The causes may be poor immobilisation, infection, poor fracture geometry and poor vascularity. The connection between fixation rigidity and non-union rate has not been fully defined and there is mixed evidence on the superiority of various plating configurations [13]. Modern systematic reviews and meta-analyses have tried to combine evidence on the available information that compares fixation methods used in mandibular angle fractures [14]. Therefore, it is of interest to compare the rate of hardware failure and non-union in reconstruction plates, single miniplates and two miniplates to fix mandibular angle fractures and also assess infection rates, malocclusion, sensory disturbance and the general complication profiles in the fixation modalities.

## Materials and Methods:

## Study design and setting:

The present retrospective comparative research was carried out at the Department of Oral and Maxillofacial Surgery, New Horizon Dental College and Research Institute, Bilaspur, Chhattisgarh, during the period between January 2024 and August 2025 and the medical records of patients who had been treated with mandibular angle fractures were reviewed. The approval of the study protocol was obtained by the Institutional Review Board and the confidentiality of the patients was ensured during the data collection and analysis.

## Sample size calculation:

The sample size was determined using initial data on the failure rates of the hardware used in miniplate and in reconstruction plates (12 per cent and 4 per cent, respectively). A minimal sample size of 55 patients per group was calculated at an alpha level of 0.05 and a statistical power of 80. A target of 60 patients per group was set to accommodate incomplete records and loss to follow-up.

## Patient selection:

The choice of medical records started with 248 patients who suffered a mandibular angle fracture. After the use of inclusion and exclusion criteria, 186 patients were included in the analysis.

[1] Inclusion criteria included: age 18 years or older, isolated mandibular angle fracture or angle fracture in combination with parasymphyseal /body fracture, treatment with open reduction and internal fixation using reconstruction plates or miniplates and a minimum 12 months follow-up with complete radiographic documentation and complete medical records including operative notes and follow-up evaluation.

[2] Exclusion criteria were: pathological fractures due to tumours or osteomyelitis, severely comminuted fractures with bone loss, condylar fractures, prior mandibular operation or hardware, incomplete medical records and follow-up less than 12 months.

## Group classification:

Patients were categorised into three groups based on the fixation technique employed:

[1] Group A (Reconstruction Plate): Patients treated with 2.4 mm locking reconstruction plates with bicortical screw fixation (n=58)

[2] Group B (Single Miniplate): Patients treated with a single 2.0 mm miniplate at the external oblique ridge according to Champy's principle (n=64)

[3] Group C (Dual Miniplates): Patients treated with two 2.0 mm miniplates at the superior and inferior mandibular borders (n=64)

## Surgical techniques:

Surgical operations were done under general anaesthesia and all the surgical operations were conducted by experienced maxillofacial surgeons. Reduction of fractures was done according to the usual procedure, with anatomical position confirmed both clinically and radiographically. To fix the plate of reconstruction, a transbuccal or lengthened intraoral procedure was applied. After exposure and reduction of the fractures, 2.4 mm locking reconstruction plates (6-8 holes) were shaped and fitted to the lateral mandibular surface. Load-bearing stability was guaranteed by bicortical screw fixation (at least 3 screws to each fragment, 12-16 mm long). Single miniplate fixation utilised an intraoral incision of the vestibular area that comprised periosteal elevation that was adequate to support the placement of the plate on the outer ridge of the oblique. The miniplate was a titanium miniplate (4-6 holes) fitted with monocortical screws according to Champy. Dual miniplate fixation was done with one plate at the superior border (external oblique ridge) and a second plate at the inferior mandibular border through transbuccal trocar or extended intraoral. Monocortical screws were used to fix the two plates. Antibiotic prophylaxis (amoxicillin-clavulanate 1g 2 times a day for 7 days), chlorhexidine mouth rinses, soft diet for 6 weeks and activity restrictions were used as postoperative protocols. The application of maxillomandibular fixation was applied as an adjunct according to the judgment of the surgeon, depending on the stability of the fracture and the patient's compliance.

## Outcome assessment:

Hardware failure (plate fracture, screw loosening, screw pullout that should be removed or revised) and non-union (failure to attain radiographic union 6 months postoperative and still have mobility or symptoms) were primary outcomes. Secondary outcomes included: surgical site infection (purulent discharge, erythema, or wound dehiscence requiring treatment), malocclusion (patient-reported versus clinically evident occlusal discrepancy), sensory disturbance (inferior alveolar nerve paresthesia continuing past 6 months) and plate removal (elective or indicated) and the overall rate of complications. Radiographic evaluation involved panoramic radiographs at 6 weeks, 3 months, 6 months and 12 months of the postoperative period. Fracture union was attributed to the formation of a bridging callus that was connected to the cortex and lacked the presence of fracture lines.

## Data collection:

The medical records were analysed to obtain demographic information (age, gender), fracture-related data (presence of a third molar, fracture displacement, related fractures), treatment variables (the use of maxillomandibular fixation, management of the third molar) and outcome data were gathered and placed in standard data collection forms.

## Statistical analysis:

The SPSS version 26.0 (IBM Corporation, Armonk, NY) was used to perform the statistical analysis. Continuous variables were expressed in mean and SD and compared using one-way ANOVA, which has a post-hoc Tukey test. The categorical variables were calculated as frequencies (percentages) and compared with each other with the help of the Chi-square test or the Fisher exact test (when using this method is appropriate). Binary logistic regression was also conducted to determine the independent predictors of hardware failure and non-union. The level of statistical significance was set to p <0.05.

## Results:

A total of 186 patients met the inclusion criteria and were included in the analysis. Demographic and clinical characteristics were comparable across treatment groups (Table 1). Mean age was 31.4 ± 10.8 years, with male predominance (82.8%). Road traffic accidents constituted the primary aetiology (54.3%), followed by interpersonal violence (28.5%) and falls (12.4%). Third molar presence at the fracture line was documented in 73.1% of cases, with extraction performed in 89.7% of these patients. Adjunctive maxillomandibular fixation was utilised more frequently in the single miniplate group (43.8%) compared to the reconstruction plate (20.7%) and dual miniplate (28.1%) groups (p=0.016). Hardware failure occurred in 14 patients (7.5%) overall, with significant differences among groups (Table 2). The reconstruction plate group demonstrated the lowest hardware failure rate (3.4%), compared to the single miniplate (12.5%) and dual miniplate (6.3%) groups (p=0.038). Plate fracture was observed exclusively in miniplate groups, with significantly higher incidence in single miniplate cases (6.3%) compared to dual miniplates (1.6%) (p=0.048). No plate fractures occurred in the reconstruction plate group. Non-union was documented in 7 patients (3.8%), with rates of 1.7%, 6.3% and 3.1% in Groups A, B and C, respectively (p=0.046). Mean time to radiographic union was significantly shorter in the reconstruction plate group (7.2 ± 1.4 weeks) compared to the single miniplate (8.6 ± 2.1 weeks) and dual miniplate (7.8 ± 1.8 weeks) groups (p=0.002). Overall complication rates differed significantly among groups, with reconstruction plates demonstrating the lowest complication burden (Table 3). Infection rates were comparable across groups (5.4% overall). Malocclusion occurred more frequently following single miniplate fixation (9.4%) compared to reconstruction plates (3.4%) and dual miniplates (4.7%), though differences approached but did not reach statistical significance (p=0.054). Sensory disturbance persisting beyond 6 months was documented in 9.7% of patients, with no significant differences among groups (p=0.468). The dual miniplate group demonstrated numerically higher rates (12.5%), potentially reflecting increased surgical dissection requirements. Operative time was significantly longer for reconstruction plate placement (78.4 ± 18.2 minutes) compared to single miniplate (52.6 ± 14.8 minutes) and dual miniplate (68.4 ± 16.4 minutes) procedures (p<0.001). Binary logistic regression identified single miniplate fixation (OR: 4.12, 95% CI: 1.28-13.24, p=0.018), displaced fractures (OR: 2.84, 95% CI: 1.12-7.18, p=0.028) and non-compliance with dietary restrictions (OR: 3.56, 95% CI: 1.34-9.48, p=0.011) as independent predictors of hardware failure.

## Discussion:

The current study illustrates that the hardware failure and non-union rates are significantly lower when using reconstruction plate fixation than when using single or two miniplate fixations in the repair of mandibular angle fractures. Such observations question the idea of traditional teaching, whereby reconstruction plates are only used in complicated patterns of fracture and may be beneficial in treating angle fractures. The failure rate of hardware in the reconstruction plate group was found to be 3.4%, which is acceptable with the already reported rate of hardware failure in miniplate fixation of 5 to 15 per cent in various series [15]. The biomechanical excellence of reconstruction plates with three-dimensional fixation by bicortical fixation is effective in counteracting functional forces that may otherwise surpass miniplate fixation [16]. This increased stability seems to be of special interest to the mandibular angle, where masticatory forces produce major stress concentrations. The observed increased hardware failure rate of single miniplate fixation (12.5%) is consistent with biomechanical studies, which show that monocortical fixation at a single location is not enough to fix the multifaceted stress patterns at the mandibular angle fractures [17]. Although the principles of Champy are still applicable to a desirable fracture configuration in compliant patients, our results indicate that single miniplate fixation is not the optimal one used with a wider range of patients [5]. Theoretical benefits of the consideration of both tension and compression zones at the fracture site are supported by the intermediate performance of the dual miniplates with a hardware failure rate of 6.3% [8]. Nevertheless, it does not imply that even the dual miniplate systems could offer adequate rigidity to ensure optimal healing in some clinical conditions because the failure rates continue to be high when compared to standard reconstruction plates [18]. Fracture of plates took place only in miniplate groups, with single miniplates proving the most vulnerable. This trend represents the decreased cross-sectional area and fatigue resistance of conventional miniplates relative to reconstruction plates [19]. The fact that the reconstruction plate group does not exhibit the plate fracture shows the increased structural integrity with load- bearing fixation. The comparable trends were observed with non-union rates of 1.7% versus 6.3% and 3.1% correspondingly in the case of reconstruction plates and single and dual miniplates. The correlation between fixation rigidity and fracture healing has received long- term research and results show increased osteogenesis when there is minimum interfragmentary motion [4]. The mechanical environment at the mandibular angle fracture seems to be optimised with reconstruction plates. Further evidence of increased healing dynamics is seen with the shorter time to radiographic union with reconstruction plates (7.2 weeks) than for miniplate groups. Accelerated union helps to minimise the vulnerability time frame, during which hardware is exposed to functional loading and this may help to minimise the failure rates [20]. The fixation techniques had different complication profiles with a general complication rate of 20.7, 34.4 and 25.0 per cent in reconstruction plates, single miniplates and dual miniplates, respectively. The increased complication rate of single miniplate fixation is the cumulative effect of the hardware failure, non-union and malocclusion [21]. There was a higher trend of malocclusion in the single miniplate group (9.4%), but the difference was not statistically significant. Poor fracture stability can allow the occurrence of micro-displacement during healing and lead to minor discrepancies in the occlusion, which have an effect on the functional outcome [11].

The frequency of sensory disturbances was similar in each group, with the highest values numerically in that of the dual miniplate configuration. The inferiority of border access necessitated by both dual miniplate and reconstruction plate procedures theoretically results in risk to the marginal mandibular nerve and the mental nerve [22]. Nonetheless, it seems that this risk can be eliminated with the help of careful surgical technique, as the sensory outcomes are acceptable in all groups. A possible drawback of this technique is the time-consuming nature of the process of plate insertion during reconstruction. The long operative time creates more exposure to anaesthesia and can affect the use of healthcare resources [23]. Nevertheless, the extra surgical investment seems to be reasonable when it is evaluated with the help of lower reoperation rates and better outcomes. A more intensive use of adjunctive maxillomandibular fixation in the single miniplate group represents an awareness of biomechanical constrained nature of the method. In cases of low fixation rigidity, surgeons seem to offset it by using supplemental immobilization but this would create an extra patient burden and compliance issue [13]. Regression analysis proved the fixation technique to be an independent predictor of hardware failure, where single miniplate fixation was fourfold more likely to predict it than reconstruction plates. Patient compliance was also introduced as a considerable modifier where adherence to the treatment in the postoperative settings is vital to the treatment outcomes [14]. Findings should be viewed in the framework of the limitations of the study. The retrospective design presents a possible selection bias, where the surgeons will choose to use the reconstruction plates in more difficult cases. Nevertheless, there was no significant difference between the baseline characteristics across groups, indicating that there was reasonable balance in case complexity [24]. Modern advancements in plating technology, such as locking miniplate systems and 3D plates, have the potential to affect outcome comparison in future research. These novel designs have superior biomechanical features and yet have minimally invasive surgical techniques [25]. The issue of cost-effectiveness is something worth considering when choosing a treatment method. Although reconstruction plates are more expensive in terms of hardware, there may be less reoperation and better outcomes, which can offset the initial expenditure. Extensive economic analyses that include direct and indirect costs would guide the decisions about resource distribution.

## Conclusion:

We show that reconstruction plate fixations give much better results than single or dual miniplate designs in mandibular angle fractures and the hardware failure rates are 3.4 and 12.5 percent and the non-union rates are 1.7 and 6.3 per cent, respectively. The above biomechanical stability provided by load-bearing reconstruction plates translates into decreased fracture healing dynamics, decreased revision surgery needs and decreased number of overall complications. Although a longer operating time is a consideration, this investment seems to be worth the health care professionals. Although technically simple, single miniplate fixation exhibits unacceptably high rates of hardware failure, which can justify the re-evaluation of the widespread use of such a method. The fixation with dual miniplates offers intermediate results, but these are still inferior compared to the fixation of plate reconstruction.

## Figures and Tables

**Table 1:** Baseline demographic and clinical characteristics

**Parameter**	**Group A (Reconstruction) n=58**	**Group B (Single Miniplate) n=64**	**Group C (Dual Miniplates) n=64**	**p-value**
Age (years), mean ± SD	32.1 ± 11.2	30.8 ± 10.4	31.2 ± 10.9	0.782
Gender (Male/Female)	48/10	53/11	53/11	0.968
Etiology				0.645
- Road traffic accident	30 (51.7%)	36 (56.3%)	35 (54.7%)	
- Interpersonal violence	18 (31.0%)	17 (26.6%)	18 (28.1%)	
- Falls	7 (12.1%)	8 (12.5%)	8 (12.5%)	
- Sports/Others	3 (5.2%)	3 (4.7%)	3 (4.7%)	
Third molar present	42 (72.4%)	48 (75.0%)	46 (71.9%)	0.912
Fracture displacement (>5mm)	24 (41.4%)	26 (40.6%)	28 (43.8%)	0.924
Associated mandibular fracture	22 (37.9%)	24 (37.5%)	26 (40.6%)	0.912
Adjunctive MMF used	12 (20.7%)	28 (43.8%)	18 (28.1%)	0.016*
Time to surgery (days), mean ± SD	3.2 ± 1.8	2.9 ± 1.6	3.0 ± 1.7	0.584
Follow-up (months), mean ± SD	18.4 ± 5.2	17.8 ± 4.8	18.1 ± 5.0	0.762
*Statistically significant (p<0.05);
MMF: Maxillomandibular Fixation

**Table 2:** Primary outcomes - hardware failure and non-union rates

**Outcome**	**Group A (Reconstruction) n=58**	**Group B (Single Miniplate) n=64**	**Group C (Dual Miniplates) n=64**	**Total n=186**	**p-value**
Hardware Failure	2 (3.4%)	8 (12.5%)	4 (6.3%)	14 (7.5%)	0.038*
- Plate fracture	0 (0%)	4 (6.3%)	1 (1.6%)	5 (2.7%)	0.048*
- Screw loosening	1 (1.7%)	3 (4.7%)	2 (3.1%)	6 (3.2%)	0.612
- Screw pullout	1 (1.7%)	1 (1.6%)	1 (1.6%)	3 (1.6%)	0.998
Non-Union	1 (1.7%)	4 (6.3%)	2 (3.1%)	7 (3.8%)	0.046*
Time to Union (weeks)	7.2 ± 1.4	8.6 ± 2.1	7.8 ± 1.8	7.9 ± 1.8	0.002*
Revision Surgery Required	2 (3.4%)	6 (9.4%)	3 (4.7%)	11 (5.9%)	0.034*
*Statistically significant (p<0.05)

**Table 3:** Secondary outcomes and complications

**Complication**	**Group A (Reconstruction) n=58**	**Group B (Single Miniplate) n=64**	**Group C (Dual Miniplates) n=64**	**Total n=186**	**p-value**
Surgical site infection	3 (5.2%)	4 (6.3%)	3 (4.7%)	10 (5.4%)	0.892
Malocclusion	2 (3.4%)	6 (9.4%)	3 (4.7%)	11 (5.9%)	0.054
Sensory disturbance (>6 months)	6 (10.3%)	4 (6.3%)	8 (12.5%)	18 (9.7%)	0.468
Wound dehiscence	2 (3.4%)	3 (4.7%)	2 (3.1%)	7 (3.8%)	0.872
Plate removal (elective)	4 (6.9%)	8 (12.5%)	6 (9.4%)	18 (9.7%)	0.558
Any complication	12 (20.7%)	22 (34.4%)	16 (25.0%)	50 (26.9%)	0.046*
Mean hospital stay (days)	2.8 ± 1.2	2.4 ± 0.9	2.6 ± 1.0	2.6 ± 1.0	0.124
Operative time (minutes)	78.4 ± 18.2	52.6 ± 14.8	68.4 ± 16.4	66.2 ± 18.6	<0.001*
*Statistically significant (p<0.05)
